# Understanding Cognitive Deficit After Subarachnoid Hemorrhage: A Memory Focused Approach

**DOI:** 10.7759/cureus.11513

**Published:** 2020-11-16

**Authors:** Michael Alfonso, Saba Aftab, Tariq Hamadneh, Nazleen Sherali, Nicholas Tsouklidis

**Affiliations:** 1 Medicine, Universidad del Rosario, Bogota, COL; 2 Medicine, California Institute of Behavioral Neurosciences & Psychology, Fairfield, USA; 3 Medicine, Hamdard College of Medicine and Dentistry, Karachi, PAK; 4 Ophthalmology, The Third Affiliated Hospital of Southern Medical University, Guangzhou, CHN; 5 Ophthalmology, California Institute of Behavioral Neurosciences & Psychology, Fairfield, USA; 6 Medicine, Liaquat University of Medical and Health Sciences, Jamshoro, PAK; 7 Health Care Administration, University of Cincinnati Health, Cincinnati, USA; 8 Medicine, Atlantic University School of Medicine, Gros Islet, LCA

**Keywords:** aneurysmal subarachnoid hemorrhage, memory loss, memory impairment, cognitive deficit, outcome

## Abstract

Aneurysmal subarachnoid hemorrhage (aSAH) is a prevalent condition affecting a large portion of the population, many of them still in productive ages. Memory impairment is a common factor amongst those patients. Memory exerts a pivotal role in productivity. That is why it is important to understand how it can be affected in post-aSAH patients. There are certain areas most affected in cases of memory disturbances, as well as its functional connections with crucial cerebral regions. Active research on functional magnetic resonance and diffusion tension imaging is used to identify compromised areas within the brain. There are suggested factors regarding poor performance, such as cerebrospinal fluid drainage and new infarction areas, which should be addressed properly to benefit these patients and simultaneously help them return to a productive and functional life.

## Introduction and background

Spontaneous subarachnoid hemorrhage (SSH) is a pathology related to extravasation of blood into the subarachnoid space. It generally occurs after extravasation of blood from some intracranial blood vessels that are prone to leak [1]. Those vessels can be depicted mainly as cerebral aneurysms and arteriovenous malformations. The pathophysiology described behind the rupture of an aneurysm usually occurs in association with the presence of comorbidities such as hypertension, tobacco smoking, sedentary lifestyle, and so on. After one of these aneurysms burst, blood passes through the blood-brain barrier, producing disturbances on self-regulatory mechanisms, electrolytes, abnormal pressure upon specific areas, which in general leads to the neurologic symptoms showed in these patients [2]. The pathognomonic characterization described in SSH includes the worst headache ever, but it is understood that a wide variety of symptoms may be displayed, such as dizziness, diplopia, sensory or motor disturbance, seizures, and memory disturbances, amongst others [3].

After subarachnoid hemorrhage, the description of memory disturbance has been presented since the XX century in cases that showed Korsakoff syndrome-like features. It was noted that one of the main characteristics in the clinical picture was memory impairment; for example, it is easy to find that after the stroke has presented, the patient can present amnesia [4]. It is important to note that memory and cognitive evaluation might be better analyzed in the less severe cases which can respond to clinical evaluation, such as those classified in Hunt and Hess (H-H) I-III [5], and after the patient has been treated through aneurysm clipping or embolization depending on certain variables [6]. As mentioned, memory loss can present after the clinical picture has started. That is why this article aims to clarify how memory is affected and why it occurs. Given that memory has a fundamental role in life and its preservation has a huge impact on productivity. It is important to call attention to the pathophysiological process behind memory compromise in SSH patients to allow further research focused on finding a better approach to this issue, and at the same time facilitating memory rehabilitation.

In addition, this article also focuses on finding those functional mechanisms affected in memory disturbance after SSH. We will analyze two different settings, the first of which presents immediately after the stroke, and the second involves cognitive and memory alterations in patients after being treated by aneurysm clipping and/or embolization. The differentiation between onset and follow up after treatment is due to primary mechanisms being activated initially, and in the latter, other circumstances result in various outcomes, such as the presence of vasospasm and delayed cerebral ischemia [1].

## Review

Methods

A review was conducted utilizing the following databases: PubMed®, ResearchGate, Google Scholar. The keywords used were "subarachnoid hemorrhage", "outcome", "memory", and "memory loss". The initial research showed more than 50 papers. The following inclusion criteria were applied: studies conducted between 2016 and 2020; studies published in the English language; studies conducted on the human subjects. After inclusion criteria were applied, 11 peer-reviewed studies satisfied the requirements and quality conditions.

Results

The selected studies showed distinctive results on memory batteries performed on patients after subarachnoid hemorrhage, showing a statistically significant correlation between memory compromise and subarachnoid hemorrhage [7]. A study conducted in Norway evaluating subarachnoid hemorrhage in the acute phase showed that delayed memory was also affected [8]. Several authors reported specific areas like the medial temporal lobe memory system and the cortical nodes of the default mode network in which the functional connectivity strength was reduced, which is why it is thought to be related to memory disturbances [9]. In another study, it was found that one of the main complaints, even up to five years post-SAH was memory impairment [10]. Others have studied specific anatomical structures using diffusion tensor imaging, a technology relevant in uncovering molecular changes. Those studies found compromise at the levels of the hippocampus, Papez circuit, and fornix contributing to the related memory disturbances [11]. Those patients could develop compensatory changes as enhanced activity in periventricular areas, which correspond to visual working tasks [12]. It is also highly important to differentiate clipping and coiling groups, given that the clipping group has been related to worse memory outcomes [13]. Remarkably, both groups are affected. In fact, one of the selected studies determined that performance scored below the 10th percentile in almost 50% of patients [14]. The results of these studies are depicted in Table 1.

**Table 1:** Studies highlighted in our review SAH - subarachnoid hemorrhage

Study	Location	Study period	Samples	Conclusion
Burke et al. [7] (meta-analysis)	Northern Ireland	2018	248	There was a significant association (p<0.01) with a weak effect on working memory.
Nordenmark et al. [8]	Norway	2012	51	In the acute phase: delayed memory is most affected. Cerebrospinal fluid and new infarctions might be related to poor cognitive outcomes.
Su et al. [9]	China	2018	50	It was observed reduced functional connectivity strength (FCS) between the medial temporal lobe memory system (MTL) and cortical nodes of the default mode network (DMN).
Persson et al. [10]	Sweden	2014-2015	26	Five years post SAH, it was noted that memory was one of the key aspects with lower punctuation.
Cho and Jang [11] (mini-review)	South Korea	1990-2019	185	Diffusion tensor imaging showed compromise in fornix, hippocampus, and Papez circuit.
Zaki et al. [13] (review)	USA	2003- 2016	415	The clipping had reduced memory compared to coiling patients.
Buunk et al. [14]	Netherlands	2009- 2019	71	Memory impairment was located below the 10th percentile in almost half the population.

Discussion

Cerebral aneurysms are lesions that have been reported with a prevalence of up to five percent of the population, bearing a huge amount of morbidity and mortality [3]. A wide variety of studies have been conducted to understand the pathological process that leads to neurological impairment, as well as studies to address the best favorable treatment in a given context to prevent mainly neurological negative outcomes.

One of the major neurologic functions that can be affected by a SAH is memory. It was described in the past as one possible symptom of this disease [4]. There are two types of memory systems: declarative memory, which allows recalling information and then proceeding to utilize this recalled information, and non-declarative memory related to learned skills. Declarative memory is an important system that retrieves memories to the conscious mind and expresses it by language; it is associated with the acquisition and consolidation of short-term memory and has been linked to specific cerebral areas like the hypothalamus and closely related regions [15].

There is a broad range of symptoms that can present after a SAH has occurred. One of the most common is headache, traditionally described as the most severe of one's life. Other sets of symptoms include vomiting, neck stiffness, loss of consciousness, seizures, confusion, eye movement compromise, limb weakness, or paralysis, among other focal neurologic deficits [16].

When subarachnoid hemorrhage is diagnosed, it is highly probable to suffer from various other complications. These symptoms can be neurologic or systemic in origin. Neurologic complications include rebleeding, vasospasm, hydrocephalus, seizures, increased intracranial pressure, and delayed cerebral ischemia. Systemic complications include cardiovascular disease, pulmonary disease, and electrolyte disturbances [3]. All complications have to be taken into account when referring to a neurologic deficit in the aftermath so that an appropriate and opportune treatment can alter and improve prognosis positively.

Pathophysiology

Once a cerebral aneurysm ruptures, blood passes into the subarachnoid space producing a series of events. Initially, lysis of red blood cells (RBC) results in oxyhemoglobin being released and eventually leading to the production of reactive oxygen species (ROS) [17]. Byproducts of lysed RBCs will act as danger-associated molecular patterns (DAMPS) that can be recognized by toll-like receptor 4 (TLR-4). Subsequently, leukocytes and microglia are recruited. This generates the induction of pro-inflammatory molecules that enhance the inflammatory response [18]. Another additional factor contributing to this process is the primary lesion of the endothelium that will activate the coagulation cascade and platelet aggregation, causing an increase in inflammation. This inflammation is believed to be responsible for vasospasm complications and likely will contribute to the final neurologic outcome [19].

Aside from the molecular process, a hemodynamic change also occurs. Cerebral blood flow (CBF) decreases in the first days. Another important factor to point out is that intracranial pressure (ICP) arises, with the issue of an altered cerebral auto-regulation, worsening the already adverse cellular environment, and facilitating processes like edema, ischemia, and cell death [2].

Memory Impairment

Regarding cognitive compromise, studies have been conducted on how memory and other executive functions are affected in the acute phase post-SAH. Nordenmark et al. studied 51 patients who had SAH. They organized patients into two groups: poor and good cognitive function based on their global cognitive impairment index. In due course, studies showed that delayed memory was the most affected function in both groups; and as medical predictors, they found that acute hydrocephalus and new cerebral infarction increase the odds of poor cognitive function [8].

Su et al. studied anatomical changes in patients after a long-term aSAH. Functional MRI in resting-state to patients and controls was conducted. Additionally, they obtained results from memory tests (auditory verbal learning test and Rey-Osterrieth Complex figure). The study results suggest that some changes can be related to memory disturbances in this category of patients. Those changes are abnormal activity mainly located in the parahippocampal gyrus, left inferior temporal gyrus, and left thalamus; another factor they described was functional connectivity strength ([FCs] related to interregional cooperation noted in cerebral fMRI) and its positive correlation with memory performance. FCs would be reduced between the left parahippocampal gyrus and left inferior parietal lobe, left inferior temporal gyrus, bilateral inferior frontal gyrus orbital part, and right middle frontal gyrus. There was an elevation in functional connectivity witnessed between the left thalamus and right inferior frontal gyrus orbital part, as well as with the right inferior frontal gyrus opercular part [9]. 

In terms of long-term cognitive impairment, Burke et al. studied patients who underwent aSAH and were treated via coiling [7]. This study suggested that there may be an effect on working memory, however weak. In addition, they found cognitive flexibility as the most compromised executive function. Persson et al. followed up with patients for five years after suffering from subarachnoid hemorrhage. This study analyzed a wide range of characteristics of each patient’s life in a cross-sectional study. Results showed that emotion was the worst scored, followed by the perception of stroke recovery and memory [10]. 

Cho and Jang reviewed the effects of brain injury associated with spontaneous subarachnoid hemorrhage. Possible regions compromised in cognitive impairment were the cingulum, fornix, hippocampus, dorsolateral prefrontal region, corticospinal tract, mammillothalamic tract, corticoreticular pathway, ascending reticular activating system, Papez circuit, optic radiation, and subcortical white matter. Additionally, these authors reported certain regions identified via diffusion tensor image in their revision, which could be related specifically to memory impairment post-SAH. These areas include the hippocampus, Papez circuit, and its related structures (thalamocortical tract, fornix, mammillothalamic tract, and cingulum). It was stated that anterior commissural fornix could be related to short-term memory, while postcommissural fornix relates to episodic memory [11].

Zaki et al. made a review of cognitive outcomes after subarachnoid hemorrhage. They covered a selection of studies regarding aSAH that, in sum, represented more than 400 patients and reported that memory seems to be affected more frequently in patients after clipping. Furthermore, they reviewed some of the different assessment tools meant for evaluating cognitive function. Concerning memory, the Wechsler memory scale and Rey Osterrieth Complex Figure test were used to assess multiple domains [13]. 

Buunk et al. studied 71 patients with SAH affecting their daily lives and overall functionality in society. Neuropsychological assessments determined that memory was affected in nearly half of the patients, but without a statistically significant p-value. Patients who had required CSF drainage, those who had most affected complex attention and executive functions, presented an increased probability of having problems returning to work [14]. 

Limitations

It is not quite clear how other pathologies, such as systemic diseases, influence cognitive outcomes within the studies. The sample population of studies is quite varied, some of them with a small sample size. The former could be related to a poor clarity of correlation with memory or any given neurologic outcome. Another factor is that the tests to evaluate memory and cognition are different in many of the studies. For that reason, results and conclusions of studies may differ from each other, which could lead to trouble when attempting to compare data and recreate the studies.

## Conclusions

Aneurysmal subarachnoid hemorrhage is a condition that affects millions of people all over the world, and it has an immense effect on cognition, consequently posing difficulties in returning to work and fulfill a functional life. Therefore, the purpose of this article was to understand how subarachnoid hemorrhage affects cognition, addressing particular attention to memory compromise. So we inquired about pathophysiological settings that may be related to the neurologic compromise seen in these patients, such as ROS release, TLR-4 activation, and associated inflammatory response. Concerning anatomical and functional regions related to memory disturbances, it was seen that diffusion tensor imaging and fMRI provide key insight about this matter. A few of the regions mentioned to have differentiated changes are the hypothalamus, frontobasal and temporal regions, Papez circuit, fornix, as well as its correlation with functionally linked structures. Additionally, some disease-related factors such as CSF drainage and new infarction may suggest poorer neurologic outcomes, possibly advocation for strategies in early detection and management of triggering conditions. Research might suggest that newly updated neuropsychological tests or perhaps disease-oriented tests should be utilized to have a more accurate evaluation of memory impairment and other executive functions, hoping that early rehabilitation and more appropriate clinical follow up in higher cognitive risk patients may help improve outcomes.
